# Synergistic nano-vaccine strategy for comprehensive activation of adaptive and innate immunity against *Staphylococcus aureus* infection

**DOI:** 10.3389/fimmu.2025.1665710

**Published:** 2025-11-03

**Authors:** Jiayue Xi, Minxuan Cui, Zhuoyue Shi, Zhuo Wan, Yufei Hou, Nan Sun, Muqiong Li, Zhengjun Ma, Yupu Zhu, Xin He, Qian Yang, Zhuojun Shi, Huifang Nie, Chaojun Song, Li Fan

**Affiliations:** 1 Shaanxi Key Laboratory of Chiral Drug and Vaccine Adjuvants, Department of Pharmaceutical Chemistry, Air Force Medical University, Xi’an, China; 2 Analysis, School of Pharmacy, Air Force Medical University, Xi’an, China; 3 Department of Hematology, Tangdu Hospital, Air Force Medical University, Xi’an, China; 4 Department of Chinese Materia Medical and Natural Medicines, School of Pharmacy, Air Force Medical University, Xi’an, China; 5 Teaching and Research Support Center, Airforce Medical Univeristy, Xi’an, China; 6 School of Life Science, Northwestern Polytechnical University, Xi’an, China

**Keywords:** PLGA nanoparticle, ESAT-6-like antigens, combination nanovaccine, comprehensive immune responses, *Staphylococcus aureus*

## Abstract

For decades, *Staphylococcus aureus* (*S. aureus*) vaccine development prioritized humoral immunity, heavily relying on recombinant protein antigens adjuvanted with aluminum (Alum), particularly in multivalent formulations. However, clinical limitations of Alum and the pressing challenge of antibiotic resistance have necessitated strategies that engage comprehensive adaptive and innate immunity. Addressing this critical gap, we engineered a biomimetic nanovaccine platform. Building on our previous finding that PLGA nanoparticles of specific stiffness effectively activate both humoral and cellular immunity, we conjugated two key ESAT-6-like virulence antigens, rEsxA and rEsxB, to stiffness-tuned PLGA nanoparticles with 25% PEG conjugation (25% NPs), designed to mimic staphylococcal capsule rigidity. We evaluated the biosafety and efficacy, both *in vitro* and *in vivo*, of single nano-vaccines (25% NPs-rEsxA or 25% NPs-rEsxB) and a vaccine combination (25% NPs-rEsxA+25% NPs-rEsxB). The combined vaccine demonstrated exceptional immunogenicity, significantly elevating antigen-specific IgG titers and inducing robust Th1/Th17-polarized cellular immunity, evidenced by 4.4-fold increases in IFN-γ and IL-17A secretion compared to Alum-adjuvanted controls. Crucially, this coordinated activation of adaptive and innate immunity conferred unprecedented protective efficacy: achieving 100% survival against a standard lethal dose (LD_100_) of *S. aureus* and 80% survival against a doubled lethal challenge (2×LD_100_)-outcomes substantially surpassing all controls. Our findings establish that dual-antigen targeting combined with biomimetic nanoadjuvants overcomes the limitations of traditional vaccines by holistically activating humoral, cellular, and innate immune responses, providing a potent strategy against invasive *S. aureus* infections, particularly relevant for combating drug-resistant strains.

## 1 Introduction

*Staphylococcus aureus* (*S. aureus*) represents a formidable and escalating global health burden. Beyond causing diverse life-threatening infections-including skin/soft tissue infections, bacteremia, pneumonia, endocarditis, and sepsis-its relentless development of multidrug resistance poses critical challenges (1, 2). The emergence of methicillin-resistant *S. aureus* (MRSA) has transformed this pathogen into a leading cause of antimicrobial resistance-related mortality, with recent data indicating nearly 100,000 MRSA-attributable deaths in 2021 alone (3, 4). This urgency prompted the WHO to designate MRSA a top-priority “deadly drug-resistant bacterium” in 2024. As traditional antibiotics lose efficacy against resistant strains, effective prophylactic vaccines represent an urgent unmet need. However, achieving sterilizing protection against *S. aureus* requires multi-faceted immunity beyond humoral responses alone, engaging robust cellular and innate defenses.

Numerous studies have investigated diverse *S. aureus* virulence factors as potential vaccine targets, including staphylococcal protein A (SpA), manganese transporter C (MntC), the ESAT-6 secretion system (Ess), and α-hemolysin (Hla) (5–10). To date, only two *S. aureus* vaccines have completed Phase III clinical trials: StaphVax (a bivalent CPS5/CPS8 conjugate vaccine) and V710 (targeting the single protein antigen iron-regulated surface determinant B (IsdB)) (1, 3, 11, 12). Both trials failed. These failures were largely attributed to insufficient antigenic breadth (targeting too few virulence mechanisms) and limitations of traditional adjuvant strategies. However, encouraging progress is emerging. A pentavalent vaccine (rFSAV), incorporating Hla, SpA, SEB, IsdB, and MntC, is currently under evaluation in Phase III trials based on promising safety and immunogenicity data (13, 14). The rFSAV employs a multi-target “cocktail” strategy designed to disrupt multiple stages of bacterial infection and utilizes alum as its adjuvant to enhance specific immune responses (14). These developments underscore that effective *S. aureus* vaccines likely require combining multiple antigenic components with novel adjuvants.

The Early Secretory Antigenic Target-6 (ESAT-6) secretion system (Ess) offers a compelling focus. Secreted proteins rEsxA and rEsxB are small, highly homologous acidic virulence factors critical for abscess formation, neutrophil lysis, and systemic dissemination (15, 16). Pioneering work by Zhang et al. demonstrated that rEsxA- and rEsxB-targeting subunit vaccines elicit protective Th1/Th17-biased immunity in murine models (17). Su et al. further revealed their synergistic capacity to activate robust TLR2/TLR4 signaling-a gateway to innate immunity-solidifying their rationale as dual targets addressing multiple infection stages (18).

**Figure 1:**
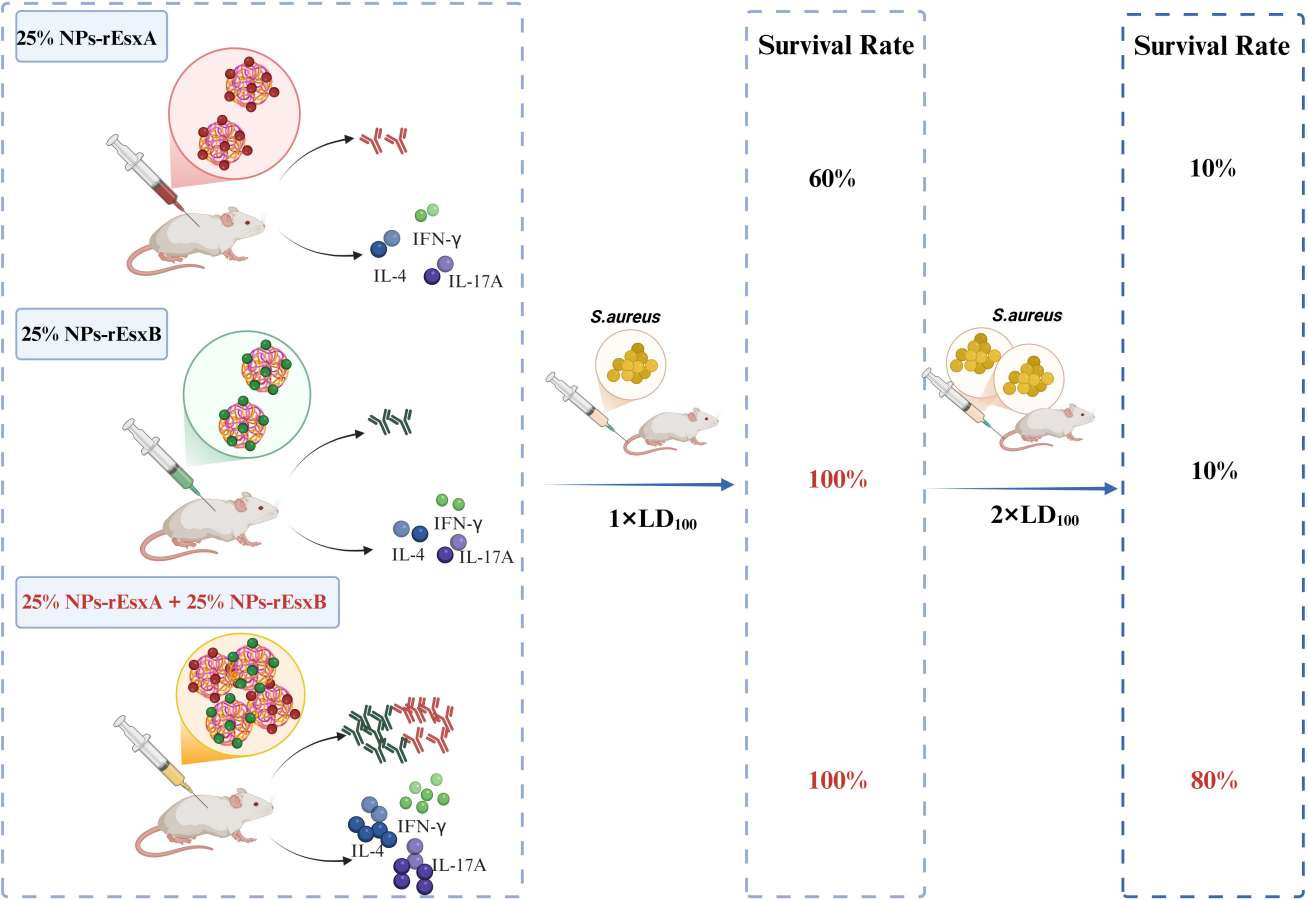
Schematic illustration of the superior immune efficacy of combined nanovaccines. Co-administration of 25% NPs-rEsxA and 25% NPs-rEsxB elicits enhanced antibody titers and Th1/Th17 cytokine production, contributing to 100% survival against standard lethal dose and 80% survival at doubled lethal dose of *S. aureus* ATCC 25923. Created in BioRender (https://BioRender.com).

Building on this foundation, our group previously established PLGA nanoparticles as superior adjuvants to Alum (19, 20). These nanoparticles co-activate potent humoral and cellular immunity against *S. aureus* through enhanced antigen stability, sustained release kinetics, and efficient APC uptake. Crucially, we discovered that modulating nanoparticle stiffness to mimic the mechanical properties of the staphylococcal capsule profoundly enhances adjuvant activity. Leveraging this insight, we engineered a synergistic nanovaccine platform using PLGA-PEG nanoparticles with 25% PEG conjugation (25% NPs) optimized for stiffness, covalently linked to recombinant rEsxA and rEsxB antigens. We hypothesized this dual-antigen nanovaccine would elicit comprehensive immunity-encompassing high-titer neutralizing antibodies, potent Th1/Th17-polarized T-cell responses, and engaged innate immunity-surpassing Alum-based formulations. To test this, we systematically evaluated monovalent (25% NPs-rEsxA or 25% NPs-rEsxB) and combined (25% NPs-rEsxA+25% NPs-rEsxB) nanovaccines for biosafety, immunogenicity (antigen-specific IgG, IFN-γ, IL-4, IL-17A), functional bacteriolytic capacity, and protective efficacy against escalating lethal challenges with *S. aureus* ATCC 25923 in murine models (**Figure 1**). This integrated approach establishes a new anti-*S. aureus* vaccination paradigm where biomaterial design and antigen synergy converge to generate multifaceted immunity against this resilient pathogen.

## 2 Materials and methods

### 2.1 Materials

PLGA_15000_-PEG_5000_ (GA: LA = 50:50) polymer was purchased from Xi’an Ruixi Biological Technology Co., Ltd. (Xi’an, China). Isopropyl β-D-thiogalactoside (IPTG), dichloromethane (DCM), 2-morpholinoethanesulfonic Acid (MES), acetone, ampicillin (AMP), imidazole were purchased from Sinopharm Chemical Reagent Co., Ltd. N-hydroxy succinimide (NHS) and 1-ethyl-3-(3-dimethylaminopropyl) carbodiimide (EDC) were purchased from Shanghai Macklin Biochemical Technology Co., Ltd. Bovine serum albumin (BSA), 2,2′-azino-bis (3-ethylbenzothiazoline-6-sulfonic acid) (ABTS), horseradish peroxidase (HRP)-labeled goat anti-mouse IgG, and 0.45 µm sterile filters were purchased from Merck (Shanghai, China). Alhydrogel™ was purchased from Croda (UK). Ninety-six-well polystyrene plates for the enzyme-linked immunosorbent assay (ELISA) were purchased from Corning (New York, NY, USA). Enzyme-linked immunospot (ELISPOT) kits were purchased from Mabtech (Stockholm, Sweden). *Escherichia coli* (*E. coil*) BL21 (DE3) was purchased from TIANGEN BIOTECH (BEIJING) Co., Ltd. (Beijing, China). Ni-Sepharose was purchased from Cytiva (Shanghai, China). A High-Capacity Endotoxin Removal column was purchased from Xiamen Bioendo Technology Co., Ltd. (Xiamen, Chian). Bradford kits were purchased from Beijing Solarbio science&technology Co., Ltd. BALC/c mice were purchased from Air Force Millitary Universities. The *S.aureus* ATCC25923 strain was obtained from Xijing Hospital (Xi’an, China).

### 2.2 Expression and purification of rEsxA and rEsxB antigen

rEsxA and rEsxB were expressed and purified from *E.coli*. Briefly, the full-length DNA sequences of rEsxA and rEsxB were cloned to pET28a vector with a 6×His-tag. The construct was transformed into *E.coli* BL21(DE3). A single colony was amplified in 5 mL Luria-Bertani (LB) medium with AMP (100 μg ml^-1^) for 3 h at 37°C while shaking. The pre-culture was diluted 1:100 in 1 L LB medium (100 µg ml^−1^ AMP) and cultured at 180 rpm at 37°C, until OD_600_ ~ 0.6. Protein expression was induced by adding IPTG, and cultivation continued under the same conditions (37°C, 180 rpm) for 4 h. The culture was centrifuged and the pellet was re-suspended in PBS. The cell suspension was homogenized and centrifuged at 15,000 × g for 10 min at 4°C. The supernatant was purified by Ni-Sepharose chromatography with 100 mmol/L imidazole wash buffer. The purified protein was dialysed and finally the buffer was replaced with PBS. The endotoxins were subsequently removed from the protein solution using the EtEraser™ HP High-Efficiency Endotoxin Removal Kit. The concentration, purity and specificity of these antigens were determined by BCA assay, SDS-PAGE and Western Blot.

### 2.3 Preparation and characterization of rEsxA- and rEsxB-conjugated 25% NPs

25% NPs were synthesized using the emulsion solvent evaporation method, as described previously (19–21). Briefly, 100 mg PLGA-PEG polymer was dissolved in a mixture of 1.5 mL dichloromethane (DCM) and 1 mL acetone. This organic solution was then added to 10 mL of 5% (w/v) polyvinyl alcohol (PVA) aqueous solution. The mixture was emulsified using an ultrasonic cell disruptor for 2 min to form an oil-in-water (O/W) emulsion. The emulsion was subsequently poured into 50 mL of deionized (DI) water and stirred for 4 h at room temperature. The resulting 25% NPs were collected, filtered through a 0.45 µm membrane filter, centrifuged at 18,000 × g for 1 h at 4°C, washed three times with DI water, and lyophilized with trehalose for storage.

rEsxA and rEsxB were covalently conjugated to the 25% NPs via an acylation reaction, following our established protocol (19). 10 mg of 25% NPs were dispersed in 10 mL of 25 mmol/L 2-(N-morpholino)ethanesulfonic acid (MES) buffer. The carboxyl groups on the NPs were activated by sequentially adding 0.4 mL of 1 mol/L 1-ethyl-3-(3-dimethylaminopropyl)carbodiimide (EDC) and 0.25 mL of 1 mol/L N-hydroxysuccinimide (NHS). The mixture was then incubated at room temperature for 3 h. The activated NPs were centrifuged at 18,000 × g for 1 h at 4°C and washed three times with DI water. The activated NP pellet was resuspended in 10 mL of PBS containing either rEsxA or rEsxB at a concentration of 1 mg/mL and incubated overnight at 4°C with gentle agitation. The resulting antigen-conjugated NPs (25% NPs-rEsxA or 25% NPs-rEsxB) were collected by centrifugation at 16,500 × g for 1 h at 4°C, washed three times with DI water, and lyophilized with trehalose for subsequent use.

The morphology of 25% NPs was confirmed through scanning electron microscope (SEM) (Quattro S, ThermoFisher, Massachusetts, US). The size distribution and zeta potential of 25% NPs, 25% NPs-rEsxA and 25% NPs-rEsxB were measured by dynamic light scattering (DLS) (Delsa™ Nano, Beckman-Coulter, High Wycombe, UK). Each sample was measured in triplicate. The successful synthesis of the 25% NPs-rEsxA or rEsxB were determined by Fourier Transform infrared spectroscopy (FTIR, Thermo Nicolet IS50, Thermo Fisher Scientific, Waltham, MA, USA). The Bradford assay was applied to quantify the total antigens loaded, and the loading efficiency was calculated using the following formula:


Loading efficiency (%)=mantigens on the NPs (mg)mNPs (mg)

### 2.4 Evaluation of biocompatibility of 25% NPs-rEsxA/rEsxB *in vitro* and *in vivo*

*In vitro* cytotoxicity of the 25% NPs nanovaccines (25% NPs-rEsxA and 25% NPs-rEsxB) against L929 fibroblast cells was assessed using the CCK-8 assay. Briefly, L929 cells were seeded into 96-well plates containing complete culture medium (RPMI 1640 supplemented with 10% fetal bovine serum (FBS) and 1% penicillin-streptomycin (Pen/Strep)) and incubated for 24 h. Subsequently, cells were treated with varying concentrations of the nanovaccines (ranging from 1 mg mL^-^¹ to 7.8125 μg mL^-^¹) for 12 h. Following treatment, the cells were washed twice with PBS, and CCK-8 solution was added to each well. The plates were then incubated at 37°C for 1–4 h. The optical density (OD) at 450 nm was measured using a microplate reader. Cell viability was calculated using the following formula:


$$\text{Cell Viability}\left(\%\right)=\left({\text{OD}}_{test}-{\text{OD}}_{background}\right)/\left({\text{OD}}_{control}-{\text{OD}}_{background}\right)\times 100\%$$

*In vivo* biocompatibility of the nanovaccines was evaluated in BALB/c mice by monitoring body weight changes. Mouse body weights were recorded every seven days for a total of five measurements following the initial vaccination.

Additionally, hematoxylin and eosin (H&E) staining of major organs was performed according to a standard protocol described in our previous work. Organs were harvested from mice euthanised 42 days after the first vaccination for histological analysis.

### 2.5 Animal immunization

Female BALB/c mice (six-week-old) were obtained from the Animal Experiment Center of Air Force Medical University. All animal procedures were conducted in accordance with the National Research Council’s Guide for the Care and Use of Laboratory Animals and were approved by the Animal Care and Ethics Committee of the Fourth Military Medical University (Approval No. KY20213144-1). Negative control groups received PBS, rEsxA (12.5 μg/mouse), rEsxB (12.5 μg/mouse), or rEsxA + rEsxB (12.5 μg each antigen, total 25 μg/mouse). Positive control groups (Alum-adjuvanted) received Alum mixed with rEsxA (12.5 μg/mouse), rEsxB (12.5 μg/mouse), or rEsxA + rEsxB (12.5 μg each, total 25 μg/mouse). Test groups received 25% NPs conjugated with rEsxA (12.5 μg antigen/mouse), rEsxB (12.5 μg antigen/mouse), or rEsxA + rEsxB (12.5 μg each antigen, total 25 μg/mouse). The mice were immunized once every two weeks for a total of three immunizations, with six mice per group, and each group received a volume of 200 μL. Blood samples were collected from the tail vein on days 0, 17, and 35 for subsequent analysis.

### 2.6 Antigen-specific antibody response assay

The rEsxA and rEsxB antibody responses were detected by enzyme-linked immunosorbent assay (ELISA) on days 35 after the first immunization. Typically, rEsxA or rEsxB protein was pre-immobilized on 96-well ELISA plate (Corning) at 10 μg/mL (PBS, 100 μL/well, incubated overnight at 4°C). And The plate was then washed 5 times with PBS containing 0.1% Tween (PBST). Twofold serially diluted mice serum ranging from 1:250 to 1: 512,000 (100 μL/well) at 37°C for 1 h. The plate was washed 5 times with PBST and incubated with HRP-labeled goat anti-mouse IgG (1:3300, 100 µL/well) at 37°C for 45 min. After washing 5 times with PBST, 100 µL of ABTS solution was added to each well for 20 min at room temperature and OD value was measured using a microplate reader (Biotek SYNERGY LX Instruments, Santa Clara, CA, USA) at 405 nm. For antibody responses analysis, the area under the curve (AUC) method was more accurate than the endpoint titer (ET) method. The antigen-specific antibody titer results were represented by the mean serum ELISA AUC with the S.D. of 6 mice in each group.

### 2.7 ELISPOT assay

On day 7 after the third boost vaccination through subcutaneous (S.C.), mice were euthanized by exposure to a gradually rising concentration of carbon dioxide (CO°C), followed by cervical dislocation to ensure death. Cytokine IL-4 (representative of humoral immunity), IL-17A (representative of Th17 T cell activation) and IFN-γ (representative of cell-mediated immunity) were analyzed using a cytokine-specific enzyme-linked immunospot (ELISPOT) assay. Briefly, the ELISPOT plate (capture antibody precoated) were washed 4 times with PBS and blocked with a blocking solution (RPMI-1640 containing 10% FBS, 200 µL/well) for 30 min at room temperature. Splenocytes isolated from immunized mice were plated at a concentration of 1 × 10^6^ cells/well and induced with each antigen alone (4 µg/ml antigen) in triplicate and incubated for 24 h at 37°C. Phytohemagglutinin (PHA, 10 µg/mL) was used as a positive control. Splenocytes from naive mice stimulated with rEsxA or rEsxB, splenocytes from unstimulated mice, and RPMI 1640-treated splenocytes were used as negative controls. After washing 5 times with PBS, the detection antibodies into PBS containing 0.5% FBS (PBS-0.5% FBS) were added to each well and incubated for 2 h at room temperature, followed by streptavidin-HRP conjugate for 1 h at room temperature. And then the plates were washed as above, the color was developed with TMB solution and the spots were counted in an ELISPOT reader (Cellular Technology Limited, Cleveland, OH, USA).

### 2.8 Lethal challenge

Staphylococcus aureus strain ATCC 25923 was grown in LB medium at 37°C with shaking at 180 rpm until mid-exponential phase (OD_600_ ~ 0.6). Bacterial cells were collected by centrifugation, washed twice, and resuspended in PBS. The bacterial concentration was adjusted spectrophotometrically based on OD_600_ and verified by plating serial dilutions on LB agar for colony-forming unit (CFU) enumeration. The challenge inoculum was prepared at a target concentration of 5.26 × 10^8^ CFU/mL, which corresponded to an LD_100_ dose based on preliminary virulence assessments. On day 42 after the first vaccination, immunized mice were intravenously injected via the tail vein with 5.26 × 10^8^ CFU (1 × LD_100_) or 10.52 × 10^8^ CFU (2 × LD_100_) of S. aureus ATCC 25923. Mice were monitored for 14 days for mortality and clinical signs. A humane endpoint protocol was strictly followed, under which any mouse exhibiting either ≥20% weight loss from baseline or severe clinical symptoms indicating irreversible distress or morbidity-such as pronounced lethargy, hunched posture, or inability to access food or water, was promptly euthanized by CO°C asphyxiation to prevent undue suffering. All surviving mice were euthanized at the conclusion of the experiment.

### 2.9 Serum bactericidal test

The bacteriolytic activity of antibodies in all experimental groups was evaluated using the standard Neisser-Wechsberg bacteriolysis assay (22, 23). *Staphylococcus aureus* ATCC 25923 was cultured to OD600 ~ 0.6. The bacterial suspension was then diluted 10-fold in sterile saline. A reaction mixture was prepared by combining 0.2 mL of the diluted bacteria, 0.2 mL of 10-fold diluted serum from immunized mice, 0.2 mL of 10-fold diluted guinea pig serum (as an exogenous complement source), and 1.4 mL of sterile saline. This mixture was incubated at 37°C for 30 minutes. After incubation, appropriate dilutions were spread onto LB agar plates and incubated overnight at 37°C to enumerate viable bacteria. Finally, the number of colony-forming units of *S. aureus* was counted and the percentage of lytic bacteria was calculated as the following formula:


Percentage of lytic bacteria(%)=(1−The CFU of treated group/The CFU of control group)×100%

### 2.10 Statistical analysis

The quantitative data are expressed as mean ± standard deviation (SD). Statistical analysis was evaluated by using an unpaired two-tailed Student’s t test and log-rank (Mantel–Cox) analysis was employed to determine the statistical significance of the survival rate. Results with * *p*< 0.05, ** *p*< 0.01, *** *p*< 0.001, and **** *p*< 0.0001 were considered statistically significant.

## 3 Results

### 3.1 Design and characterizations of 25% NPs-rEsxA/rEsxB nanovaccines

**Figure 2:**
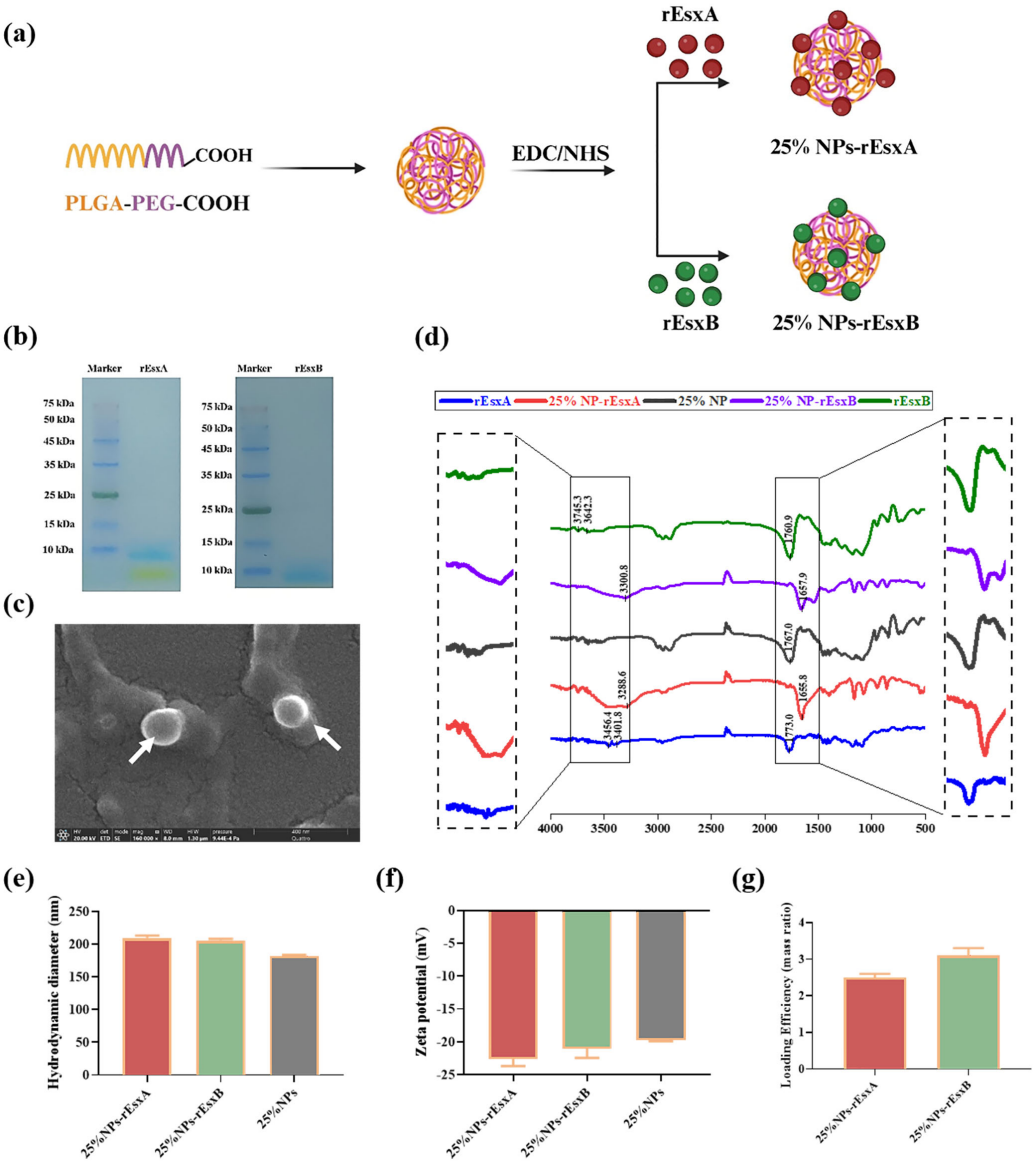
The design and characterization of 25% NPs nanovaccine. **(a)** Schematic illustration of the synthetic route for 25% NPs. **(b)** SDS-PAGE analysis of the rEsxA and rEsxB antigens. **(c)** Representative SEM image of the 25% NPs. **(d)** FT-IR spectra of rEsxA, rEsxB, 25% NPs, 25% NPs-rEsxA and 25% NPs-rEsxB. **(e)** Hydrodynamic diameter of the 25% NPs measured by DLS. **(f)** Zeta potential of the 25% NPs measured by DLS. **(g)** Loading efficiency of rEsxA and rEsxB in the 25% NPs-rEsxA or rEsxB measured by BCA.

25% NPs were synthesized as previously described, with the synthetic route schematically illustrated in **Figure 2A**. The preparation and purification of the recombinant proteins rEsxA and rExsB were carried out following the standard protocols. The rEsxA and rExsB were then loaded on the NPs surface by covalent conjugation, respectively.

SDS-PAGE analysis was employed to testify the successful preparation and purification of the rEsxA and rExsB antigen. We then examined the NPs and NPs-antigen’s morphology, size distribution and zeta potential by SEM and DLS.

As shown in **Figure 2B**, the molecular weight of rEsxA and rEsxB were 11.5 kDa, confirmed by SDS-PAGE. SEM results showed spherical morphology of the 25% NPs (**Figure 2C**). The hydrodynamic diameter of bare 25% NPs was 182.4 ± 1.2 nm (**Figure 2E**), and the zeta potential was -19.73 ± 0.2 mV (**Figure 2F**). rEsxA and rEsxB were then conjugated onto the NPs surface by amide covalent bonding, confirmed by FT-IR spectra. The amide bond peaks at 3288.6 cm^-^¹ (ν_N-H_) and 1655.8 cm^-^¹ (ν_C=O_) for 25% NPs-rEsxA, and the peaks of 3300.8 cm^-^¹ (ν_N-H_) and 1657.9 cm^-^¹ (ν_C=O_) for 25% NPs-rEsxB, confirmed the successful conjugation of rEsxA or rEsxB to the 25% NPs, respectively (**Figure 2D**).

After conjugation with rEsxA or rEsxB, the hydrodynamic diameters increased to 209.7 ± 0.6 nm (25% NPs-rEsxA) and 205.8 ± 0.8 nm (25% NPs-rEsxB), indicating that the conjugation of antigen leads to a ~20 nm size increase (**Figure 2E**). The zeta potential became more negatively after antigen conjugation (**Figure 2F**), with -22.59 ± 1.1 mV for 25%NPs-rEsxA and -21.04 ± 1.4 mV for 25%NPs-rEsxB). BCA assay revealed that the loading efficiency of both 25% NPs-rEsxA and 25% NPs-rEsxB was ~3.0% (mass ratio) (**Figure 2G**).

### 3.2 The biocompatibility of nanovaccines

**Figure 3:**
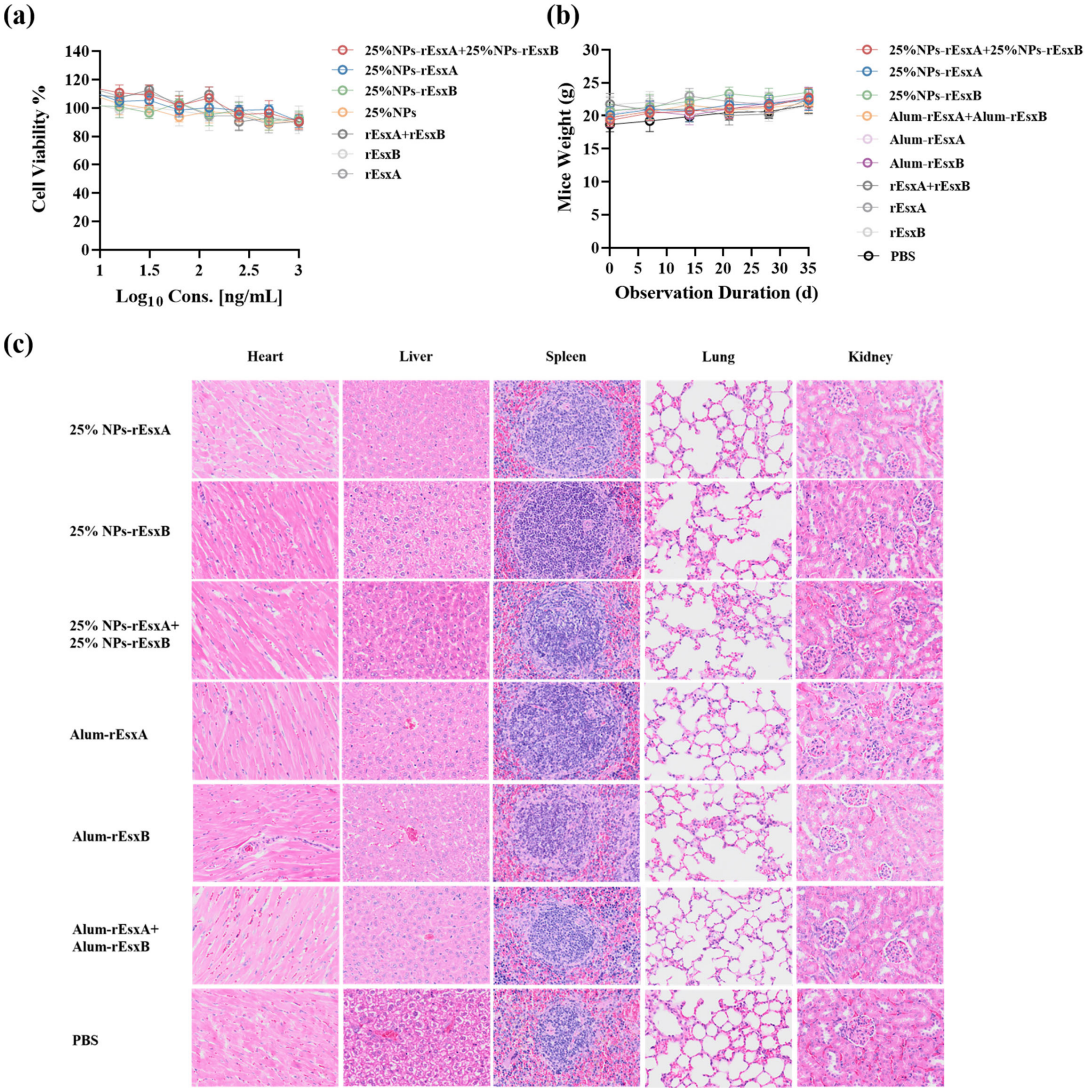
Biocompatibility evaluation of 25% NPs nanovaccines. **(a)** Viability of L929 cells exposed to nanovaccines. **(b)** Body weight dynamics in mice after subcutaneous (s.c.) nanovaccine administration. **(c)** Representative H&E-stained sections of major organs (heart, liver, kidney, lung, spleen) from nanovaccine-treated mice.

Before the efficacy evaluation of the nanovacines, 25% NPs-rEsxA, 25% NPs-rEsxB and 25% NPs-rEsxA+25% NPs-rEsxB, the biosafety issues were first addressed *in vitro* and *in vivo*. CCK-8 assays, which employed to test the cytotoxicity of the nanovaccines, demonstrated the well cell viability (>85%) in all tested groups (**Figure 3A**).

Subcutaneous administration in mice caused no significant weight changes compared to controls (**Figure 3B**). Histopathological analysis via H&E staining revealed that no lesions in major organs (heart, liver, kidney, lung, spleen) were observed in immunized mice (**Figure 3C**; **Supplementary Figure S1**).

### 3.3 The humoral immune response activation efficacy of the nanovaccines

**Figure 4:**
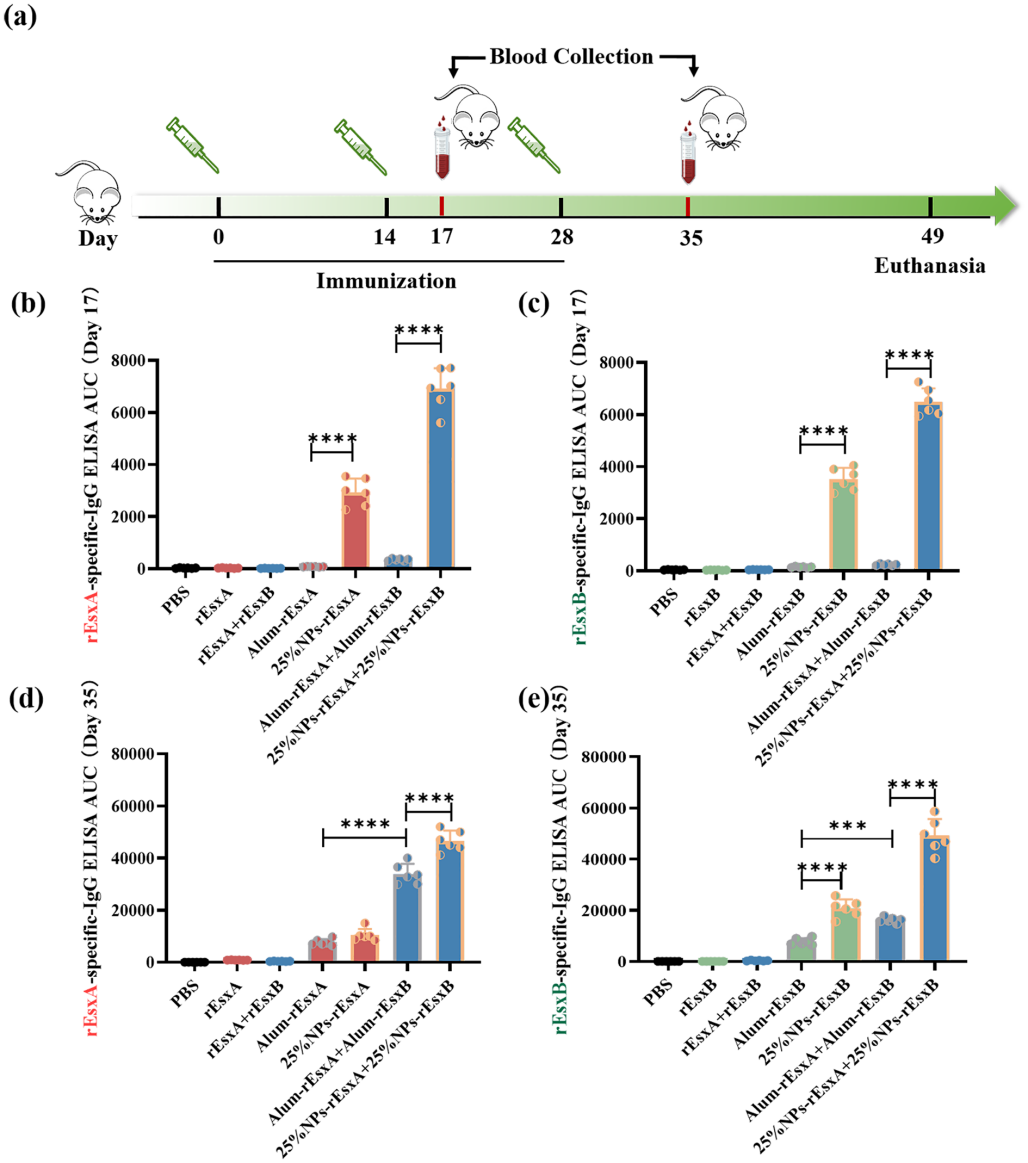
Potent humoral response to *S. aureus* antigens after S.C. vaccinations with the PLGA nanovaccines. **(a)** Illustration of the immunization protocol and the sampling time points. **(b)** rEsxA-specific serum IgG level at day 17 (n=6). **(c)** rEsxB-specific serum IgG level at day 17 (n=6). **(d)** rEsxA-specific serum IgG level at day 35 (n=6). **(e)** rEsxB-specific serum IgG level at day 35 (n=6). All data were expressed as mean ± S.D. A *p* value< 0.05 was considered as statistically significant (***p< 0.001; ****p< 0.0001).

To evaluate the humoral immunity induced by the nanovaccines, antigen-specific IgG levels were measured after immunization in all groups. Vaccination was performed according to standard protocols (prime on day 0; boosts on days 14 and 28; **Figure 4A**) via subcutaneous administration. Blood samples were collected on days 17 and 35 after the first administration. Day 17 was selected to capture the early adaptive immune response, representing a critical time window for assessing the initial immunogenicity of the vaccine prior to the dominance of memory responses. An ELISA assay was used to assess humoral immune activation. The results showed that at day 17 after the first vaccination, IgG responses in the PBS, free antigen, and Alum-adjuvanted vaccine groups were barely detectable. In contrast, the nanovaccine groups exhibited a significant increase in IgG response. Moreover, the combined nanovaccine group (25% NPs-rEsxA + 25% NPs-rEsxB) showed an approximately 1.8-fold higher IgG response compared to the single-antigen nanovaccine groups (25% NPs-rEsxA or 25% NPs-rEsxB) (**Figures 4B, C**).

By day 35 after the first vaccination, the PBS and free antigen groups remained seronegative, while the Alum-adjuvanted vaccine groups produced significant IgG levels. The nanovaccine groups showed further increases in IgG antibody levels compared to day 17. When comparing the Alum-adjuvanted vaccines and the nanovaccines, the rEsxA-specific IgG response was comparable between the two groups (**Figure 4D**). However, the rEsxB-specific IgG response was significantly higher in the nanovaccine group than in the Alum-adjuvanted group. In the combined vaccination groups (Alum-rEsxA + Alum-rEsxB and 25% NPs-rEsxA + 25% NPs-rEsxB), IgG responses to rEsxA and rEsxB were evaluated separately (**Figure 4E**). The results demonstrated that for both rEsxA- and rEsxB-specific IgG, the AUC values of the 25% NPs-adjuvanted vaccine group were significantly higher than those of the Alum group, indicating that 25% NPs serves as a more effective adjuvant for the recombinant proteins rEsxA and rEsxB.

### 3.4 The cellular immune response activation efficacy by the nanovaccines

In order to test the tendency of the immune response, ELISPOT assay was employed to study the specific cytokines, IL-4 representative for Th2, IFN-γ for Th1/CTL and IL-17A for Th17/innate immune response.

**Figure 5:**
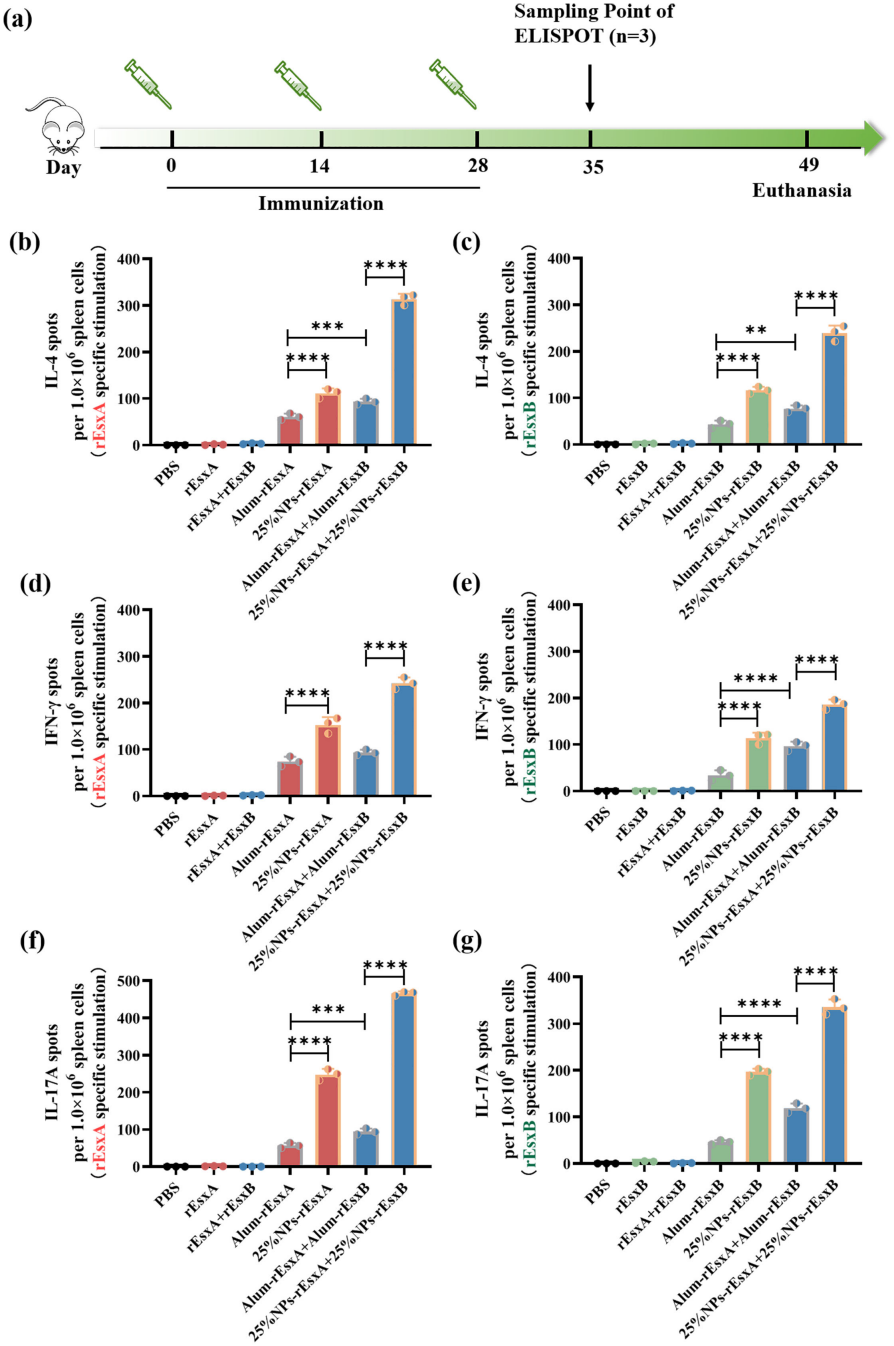
Potent humoral and cellular response to *S. aureus* antigens after S.C. vaccinations with the PLGA nanovaccines. **(a)** Illustration of the immunization protocol and the sampling time points. **(b)** IL-4 secretion by rEsxA (10 μg/mL) stimulation revealed by ELISPOT (n=3). **(c)** IL-4 secretion by rEsxB (10 μg/mL) stimulation revealed by ELISPOT (n=3). **(d)** IFN-γ secretion by rEsxA (10 μg/mL) stimulation revealed by ELISPOT (n=3). **(e)** IFN-γ secretion by rEsxB (10 μg/mL) stimulation revealed by ELISPOT (n=3). **(f)** IL-17A secretion by rEsxA (10 μg/mL) stimulation revealed by ELISPOT (n=3). **(g)** IL-17A secretion by rEsxB (10 μg/mL) stimulation revealed by ELISPOT (n=3). All data were expressed as mean ± S.D. A *p* value< 0.05 was considered as statistically significant (**p< 0.01; ***p< 0.001; ****p< 0.0001).

Firstly, for IL-4 production, though there was no significant difference of IgG titer between Alum-rEsxA and 25% NPs-rEsxA, the IL-4 spots secreted by spleen cells of the vaccinated mice in 25% NPs-rEsxA group was significantly higher than that in Alum-rEsxA ones. The results indicated that 25% NPs served as the rEsxA adjuvant could activate the IL-4 associated Th2 immune response more efficiently (**Figure 5B**; **Supplementary Figure S3**). Similarly, we compared the IL-4 spots data of rEsxB between the Alum vaccine and the 25% NPs ones. The same trend was found with the antibody titer results in **Figure 4E**. The 25% NPs was more helpful for IL-4 secretion compared with Alum adjuvant, when the vaccine antigen is rEsxB (**Figure 5C**; **Supplementary Figure S3**). It was undoubtable that in the combined vaccination batch, 25% NPs vaccines performed better than the Alum ones, either for rEsxA or rEsxB.

We also compared the IFN-γ production in all tested and control groups, in order to investigate the cellular immune response activation issues by ELISPOT assay. For either rEsxA or rEsxB specific stimulation, the IFN-γ spots in 25% NPs adjuvant vaccine groups were more than that in Alum adjuvant vaccine groups, no matter for single antigen nor antigen combination cases (**Figures 5D, E**; **Supplementary Figure S4**). The results indicated that 25% NPs served as rEsxA or rEsxB adjuvant could better stimulate the Th1/CTL immune responses than Alum adjuvant.

As a critical effector of innate immunity and a bridge to adaptive immunity, IL-17A plays a pivotal role in host defense. Similar trend was found in the IL-17A ELISPOT assay compared with the IFN-γ secretion results. For single antigen loading, 25% NPs vaccines elicited 249 (25% NPs-rEsxA) and 203 (25% NPs-rEsxB) IL-17A spots, about 4.4-fold higher than Alum ones (**Figures 5F, G**, **Supplementary Figure S5**). Moreover, for combined vaccines treatment groups, 25% NPs vaccine groups further amplified this immune response, with IL-17A production 470 and 353 spots for rEsxA and rEsxB, respectively. Except for the humoral and cellular immune response stimulation, the enhancement of innate immune response by 25% NPs adjuvant may lead to better body protection against *S. aureus* than Alum vaccines.

### 3.5 Bacteriolysis assay evaluates vaccine-induced antibody-dependent bactericidal activity

**Figure 6:**
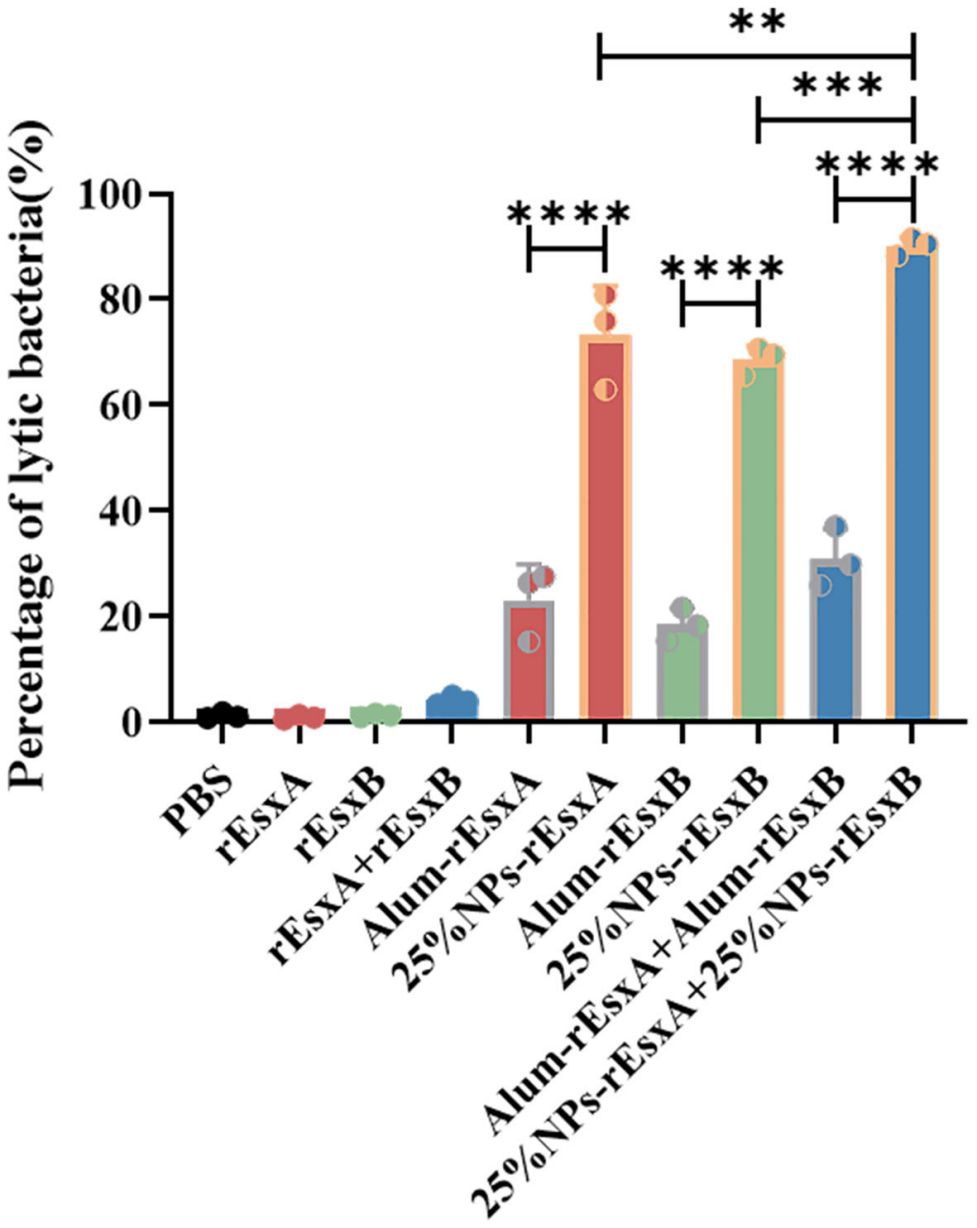
The bacteriolysis of serum antibody by S.C. vaccination (n = 3). All data were expressed as the means ± S.D. A p value < 0.05 was considered as statistically significant (** p < 0. 01; *** p < 0.001; **** p < 0.0001).

Bacteriolysis assay was performed to evaluate the antibody-dependent bactericidal activity elicited by vaccination with 25% NPs-based vaccines or Alum-adjuvanted vaccines, using free antigen and PBS as controls. As shown in **Figure 6**, antibodies from mice immunized with the 25% NPs vaccines exhibited a significantly enhanced capacity to promote bacterial clearance compared to those from the Alum group.

### 3.6 The animal protection ability against S. aureus by the nanovaccines

**Figure 7:**
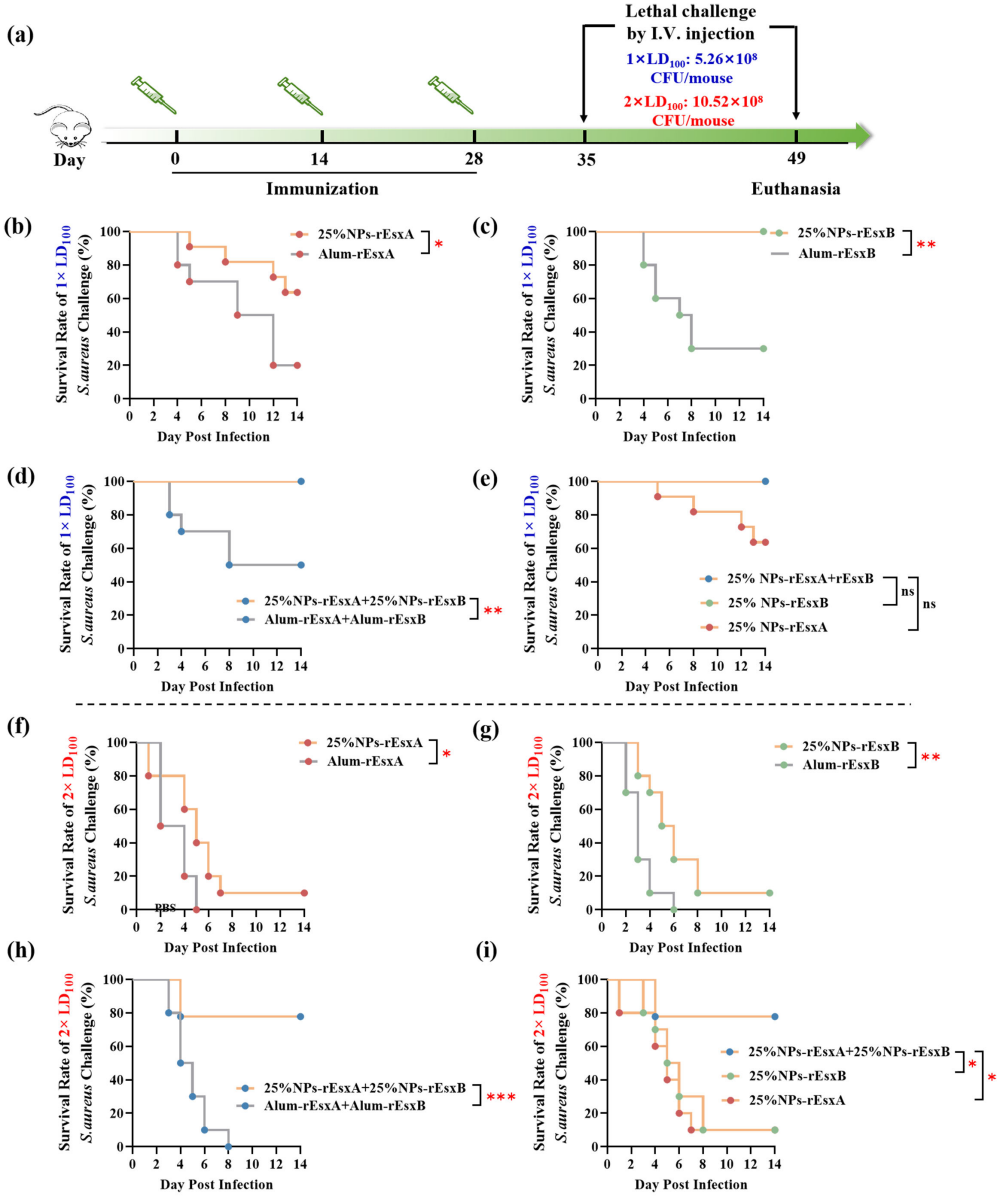
Protective efficacy of vaccinated BALB/c mice after *S. aureus* challenge. **(a)** Timeline for immunization, challenge and evaluation of protective efficacy. **(b–e)** Survival rates (n = 10) of immunized mice challenged via I.V. injection with *S. aureus* ATCC 25923 at a dose of 5.26 × 10^8^ CFU. Survival rate (n = 10) of the immunized mice challenged with *S. aureus* ATCC 25923 strain (5.26 × 108 CFU) by I.V. injection. (f-i) Survival rates (n = 10) of immunized mice challenged via I.V. injection with *S. aureus* ATCC 25923 at a dose of 10.52 × 10^8^ CFU. Survival rates were analyzed with Log-rank (Mantel-Cox) analysis. A *p* value < 0.05 was considered as statistically significant (* *p* < 0.05; ** *p* < 0.01; *** *p* < 0.001).

In order to investigate the body protection ability of the nanovaccines, we challenged the mice with the lethal dose (1×LD_100_) of *S. aureus* via vein tail administration at 35 days after the first immunization (**Figure 7A**). As respected, all mice in the PBS and free antigen groups died within 8 days (**Supplementary Figure S6**). In Alum adjuvant vaccine groups, the survival percentage was only 20% and 40% in Alum-rEsxA and Alum-rEsxB, respectively. Even in the Alum-rEsxA and Alum-rEsxB combined vaccination group, the survival percentage just reached 50% (**Figures 7B–D**). However, in 25% NPs adjuvant vaccines groups, no matter for single antigen loaded vaccine groups nor the combined vaccination group, the survival percentage was much higher than the corresponding Alum ones. Moreover, for 25% NPs-rEsxB and 25% NPs-rEsxA and 25% NPs-rEsxB combined vaccination groups, all animals survived in the lethal challenge experiment (**Figure 7E**).

Though significant differences were found in the results of antibody titer, IL-4, IFN-γ, IL-17A secretion and neutralizing capability of the antibody between the single antigen vaccine (25% NPs-rEsxB) and combine vaccine (25% NPs-rEsxA and 25% NPs-rEsxB) groups (**Supplementary Figure S2**), both of them got 100% survival percentage in the lethal challenge experiment. It was indicated by the results above that if the lethal dose increases, the protection ability between the single antigen vaccine and the combine one could be differentiated.

Thus, we further double the challenge dose of *S. aureus* to test the difference of protection ability between the single antigen loaded and the combined vaccine groups when 25% NPs served as adjuvants. Undoubtedly, the animals of all PBS and free antigen vaccination groups died out within 5 days after challenge (**Supplementary Figure S7**). Moreover, the Alum vaccine groups, either for single antigen loaded or the combined vaccines groups, the animals died out within 8 days (**Figures 7F–H**). In contrast, some of the animals in 25% NPs vaccine groups kept alive till the end of the challenge experiment. 20% and 30% of the animals in 25% NPs-rEsxA and 25% NPs-rEsxB were alive, while the survival percentage reached 80% in 25% NPs-rEsxA and 25% NPs-rEsxB combined vaccination group (**Figure 7I**).

The results of lethal challenge experiments reflected the superior body protection ability of the 25% NPs vaccines, especially for the 25% NPs-rEsxA and 25% NPs-rEsxB combined vaccination route. And these results were also consistent with the findings in the antibody titer and the cytokine secretion studies.

## 4 Discussions

*S. aureus*, particularly methicillin-resistant strains (MRSA), poses an escalating global health threat exacerbated by antibiotic resistance and the lack of effective vaccines. Although multi-antigen strategies-such as Zou et al.’s Phase III pentavalent rFSAV/Alum formulation-represent recent progress, conventional adjuvants like Alum remain constrained by Th2-biased immunity and inadequate cellular activation. Our study bridges these gaps through a dual-pronged innovation (1): rational selection of synergistic ESAT-6-like virulence factors (rEsxA/rEsxB), which target critical infection stages, including abscess formation and immune evasion; and (2) deployment of stiffness-engineered 25% NPs as biomimetic adjuvants. This strategy uniquely elicits comprehensive immunity, outperforming Alum by inducing significantly higher bactericidal antibodies, robust Th1/Th17 polarization, and unprecedented *in vivo* protection-achieving 100% survival at 1×lethal dose and 80% survival at 2×lethal dose. These results underscore the paradigm that next-generation *S. aureus* vaccines require both multi-antigen targeting and advanced delivery platforms capable of co-activating adaptive and innate immunity, as exemplified by recent biomimetic mineralized vaccines enhancing STING pathway engagement. Our 25% NPs platform further advances this paradigm by mimicking bacterial capsule stiffness, thereby enhancing dendritic cell uptake and prolonging antigen presentation.

Despite numerous *S. aureus* vaccines in clinical trials, most have failed to demonstrate significant efficacy. This may stem from the recall of non-protective immune imprints from prior *S. aureus* exposure (24, 25). While laboratory mice rarely encounter human *S. aureus* strains, up to 50% of humans are colonized or infected within two months of life, exhibiting lifelong exposure-evidenced by rising anti-*S. aureus* antibody titers in human serum. Crucially, these antibodies lack protective capacity. For instance, the V710 vaccine, targeting the immunodominant antigen IsdB, induced non-protective antibodies in *S. aureus*-immune individuals, leading to clinical failure (26). Consequently, targeting subdominant antigenic domains has emerged as a promising strategy in infectious disease models. Beyond robust antibody responses, T cell-mediated immunity is equally critical (27, 28). Here, rEsxA and rEsxB-subdominant antigens with inherent T cell immunogenicity-synergized with our adjuvant to elicit potent immune responses. A key finding of our work is the potent bacteriolytic activity observed *in vitro*. While Gram-positive bacteria like *S. aureus* are generally resistant to complement-mediated lysis due to their thick peptidoglycan layer, complement-dependent cytotoxicity (CDC) can still occur. The Cruz group demonstrated that antibodies against SPA and IsdA enhanced complement deposition on the bacterial surface, leading to killing (29). Our data are consistent with this mechanism. We hypothesized that the transient surface exposure of antigens during secretion enables antibody binding, which in turn drives complement-dependent killing. Notably, our formulation halved the antigen dose used in prior studies while maintaining efficacy. When conjugated with 25% NPs, both single nanovaccines (25% NPs-rEsxA or 25% NPs-rEsxB) and the combined formulation (25% NPs-rEsxA+25% NPs-rEsxB) induced high antibody titers and robust IFN-γ/IL-17A secretion. Through coordinated humoral, cellular, and innate immune activation, 100% survival was achieved against lethal-dose challenge. These findings highlight that comprehensive vaccine evaluation must integrate antibody titers, cellular immunity, and innate responses-not serology alone. Protective efficacy demands robustness across all three components, a principle embedded in our tripartite assessment framework.

Nevertheless, limitations must be acknowledged. Our antigen loading efficiency (~3%) remains suboptimal compared to high-performance nanovaccines. Future work should optimize conjugation chemistry or explore core-shell encapsulation to enhance payload without compromising bioactivity. Additionally, evaluation against prevalent MRSA strains (e.g., USA300) is essential to assess clinical translatability.

In summary, this study not only introduces a promising nanovaccine candidate but also establishes an evaluative framework emphasizing tripartite immune synergy, offering a roadmap for combating evolving bacterial threats.

## Data Availability

The original contributions presented in the study are included in the article/**Supplementary Material**. Further inquiries can be directed to the corresponding authors.
