# Correction: Synergistic nano-vaccine strategy for comprehensive activation of adaptive and innate immunity against *Staphylococcus aureus* infection

**DOI:** 10.3389/fimmu.2025.1740490

**Published:** 2025-11-26

**Authors:** Jiayue Xi, Minxuan Cui, Zhuoyue Shi, Zhuo Wan, Yufei Hou, Nan Sun, Muqiong Li, Zhengjun Ma, Yupu Zhu, Xin He, Qian Yang, Zhuojun Shi, Huifang Nie, Chaojun Song, Li Fan

**Affiliations:** 1 Shaanxi Key Laboratory of Chiral Drug and Vaccine Adjuvants, Department of Pharmaceutical Chemistry, Air Force Medical University, Xi’an, China; 2 Analysis, School of Pharmacy, Air Force Medical University, Xi’an, China; 3 Department of Hematology, Tangdu Hospital, Air Force Medical University, Xi’an, China; 4 Department of Chinese Materia Medical and Natural Medicines, School of Pharmacy, Air Force Medical University, Xi’an, China; 5 Teaching and Research Support Center, Airforce Medical Univeristy, Xi’an, China; 6 School of Life Science, Northwestern Polytechnical University, Xi’an, China

**Keywords:** PLGA nanoparticle, ESAT-6-like antigens, combination nanovaccine, comprehensive immune responses, *Staphylococcus aureus*

**Figures 6F, G** were wrongly cited in the following section instead of **Figures 5F, G**. A correction has been made to the section **3 Results**, *3.4 The cellular immune response activation efficacy by the nanovaccines*, paragraph 4:

“Similar trend was found in the IL-17A ELISPOT assay compared with the IFN-γ secretion results. For single antigen loading, 25% NPs vaccines elicited 249 (25% NPs-rEsxA) and 203 (25% NPs-rEsxB) IL-17A spots, about 4.4-fold higher than Alum ones (**Figures 5F, G**, **Supplementary Figure S5**)”.

**Figure 7** was erroneously cited in the following section, instead of **Figure 6**. A correction has been made to the section **3 Results**, *3.5 Bacteriolysis assay evaluates vaccine-induced antibody-dependent bactericidal activity*:

“As shown in **Figure 6**, antibodies from mice immunized with the 25% NPs vaccines exhibited a significantly enhanced capacity to promote bacterial clearance compared to those from the Alum group”.

**Figure 6** was erroneously cited in the following paragraph, instead of **Figure 7**. A correction has been made to the section **3 Results**, *3.6 The animal protection ability against S. aureus by the nanovaccines*, paragraph 1:

“In order to investigate the body protection ability of the nanovaccines, we challenged the mice with the lethal dose (1×LD100) of *S. aureus* via vein tail administration at 35 days after the first immunization (**Figure 7A**). As respected, all mice in the PBS and free antigen groups died within 8 days (**Supplementary Figure S6**). In Alum adjuvant vaccine groups, the survival percentage was only 20% and 40% in Alum-rEsxA and Alum-rEsxB, respectively. Even in the Alum-rEsxA and Alum-rEsxB combined vaccination group, the survival percentage just reached 50% (**Figures 7B–D**). However, in 25% NPs adjuvant vaccines groups, no matter for single antigen loaded vaccine groups nor the combined vaccination group, the survival percentage was much higher than the corresponding Alum ones. Moreover, for 25% NPs-rEsxB and 25% NPs-rEsxA and 25% NPs-rEsxB combined vaccination groups, all animals survived in the lethal challenge experiment (**Figure 7E**)”.

**Figure 6** was erroneously cited in the following paragraph, instead of **Figure 7**. A correction has been made to the section **3 Results**, *3.6 The animal protection ability against S. aureus by the nanovaccines*, paragraph 3:

“Thus, we further double the challenge dose of *S. aureus* to test the difference of protection ability between the single antigen loaded and the combined vaccine groups when 25% NPs served as adjuvants. Undoubtedly, the animals of all PBS and free antigen vaccination groups died out within 5 days after challenge (**Supplementary Figure S7**). Moreover, the Alum vaccine groups, either for single antigen loaded or the combined vaccines groups, the animals died out within 8 days (**Figures 7F–H**). In contrast, some of the animals in 25% NPs vaccine groups kept alive till the end of the challenge experiment. 20% and 30% of the animals in 25% NPs-rEsxA and 25% NPs-rEsxB were alive, while the survival percentage reached 80% in 25% NPs-rEsxA and 25% NPs-rEsxB combined vaccination group (**Figure 7I**)”.

The original version of this article has been updated.

