# Supplementary material for: Synergistic nano-vaccine strategy for comprehensive activation of adaptive and innate immunity against Staphylococcus aureus infection
Source: Front Immunol. 2025 Nov 3;16:1665710. doi: 10.3389/fimmu.2025.1665710 (PMC12620494; doi:10.3389/fimmu.2025.1665710)
Supplement: Supplementary file 1 [file Table1.docx]

Supplementary Material

# Supplementary Figures


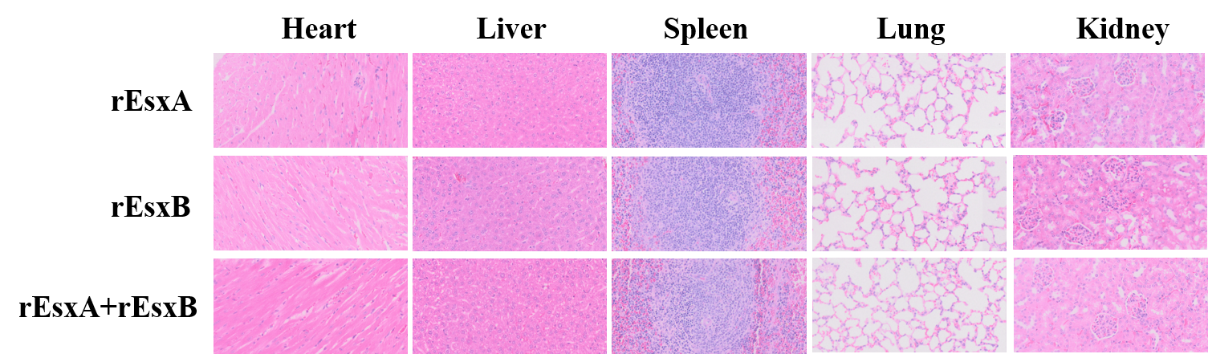


**Supplementary Figure 1.** H&E staining of major organs of the mice treated by rEsxA, rEsxB and rEsxA+rEsxB.


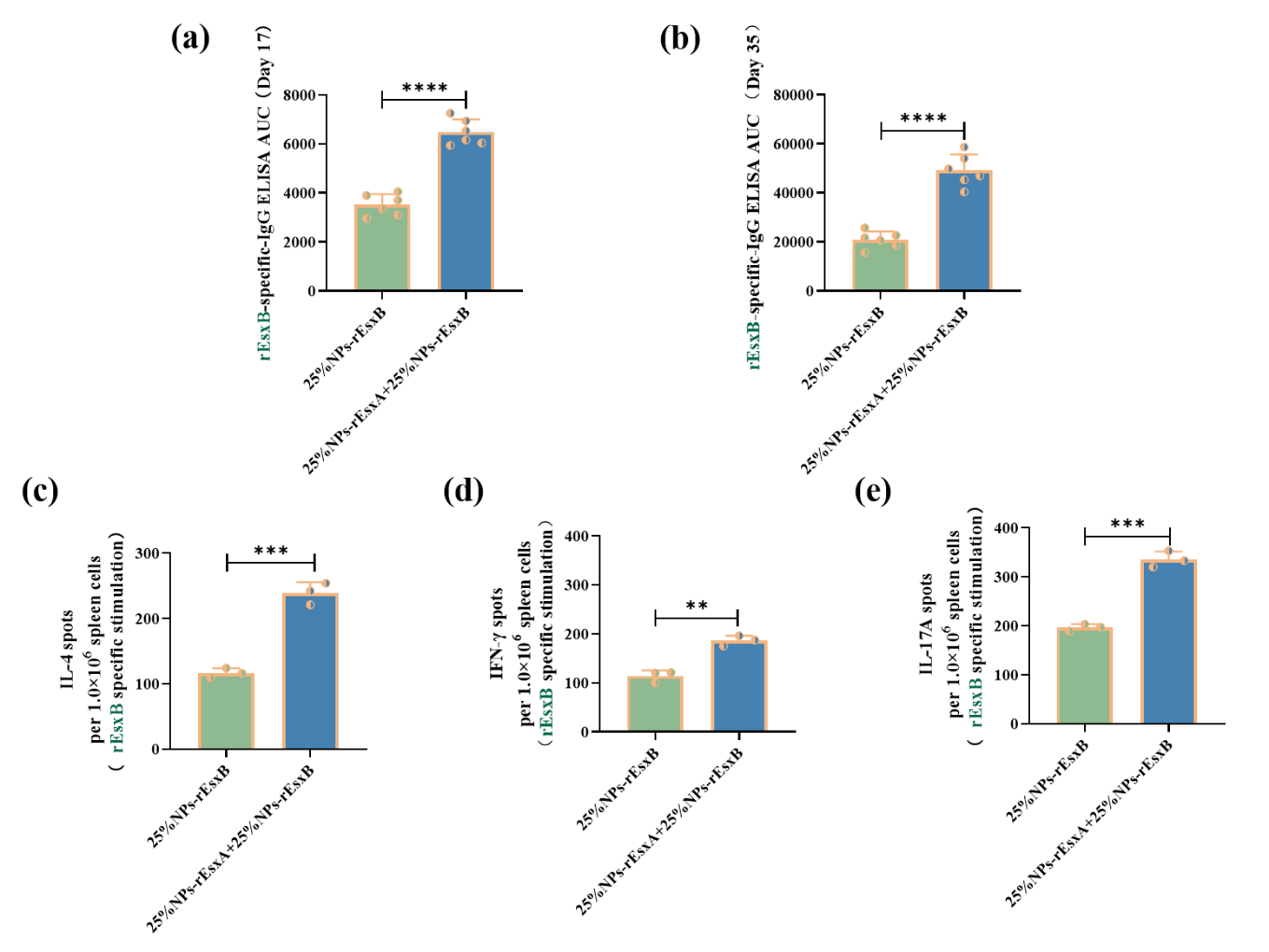


**Supplementary Figure 2.** Comparison of humoral and cellular immune responses induced by single-antigen vaccine **(**25% NPs-rEsxB**)** and combined vaccine **(**25% NPs-rEsxA + 25% NPs-rEsxB**)** groups. (**a**) Anti-rEsxB antibody titers at day 17. (**b**) Anti-rEsxB antibody titers at day 35. (**c**) The IL-4 spots secretion. (**d**) The IFN-γ spots secretion. (**e**) The IL-17A spots secretion.


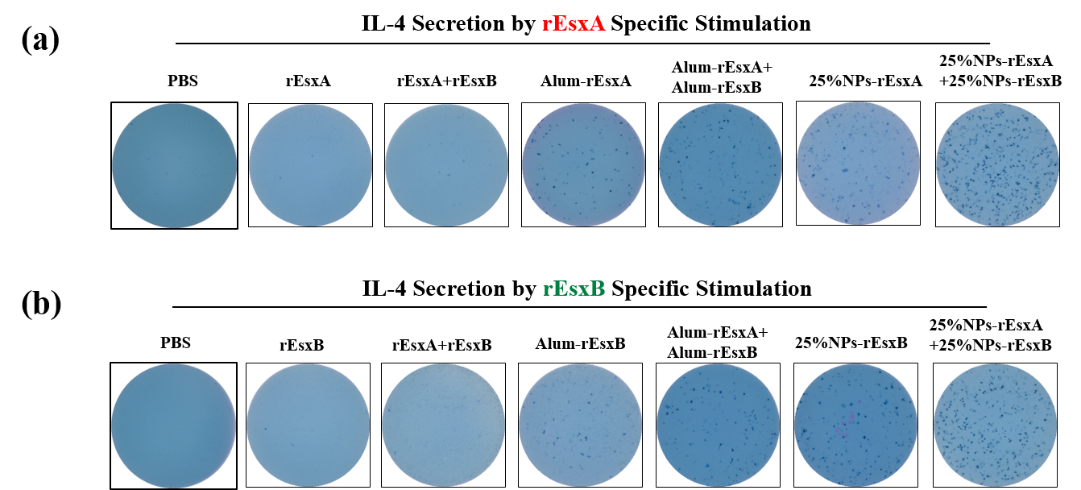


**Supplementary Figure 3.** Representative images of wells with IL-4-producing splenocytes are shown.


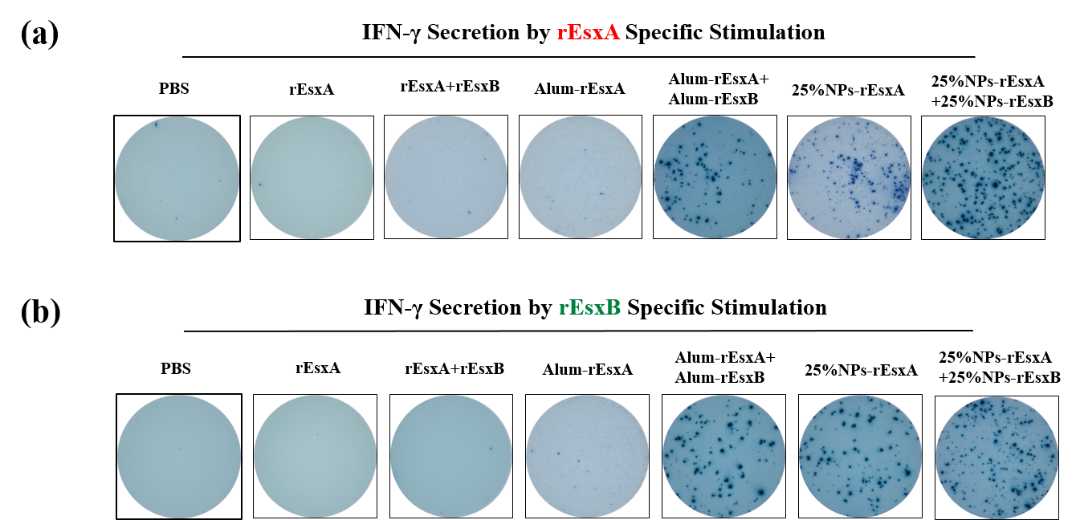


**Supplementary Figure 4.** Representative images of wells with IFN-γ-producing splenocytes are shown.


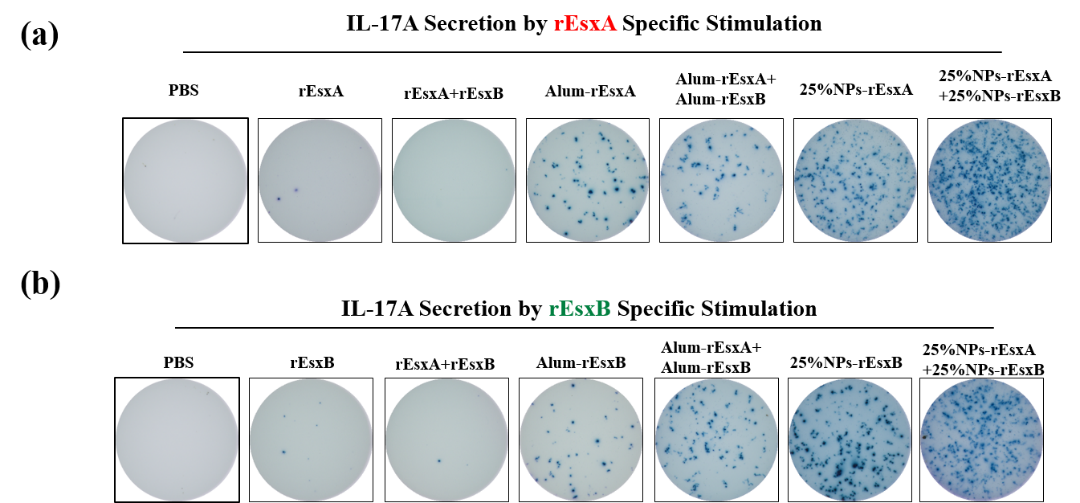


**Supplementary Figure 5.** Representative images of wells with IL-17A-producing splenocytes are shown.


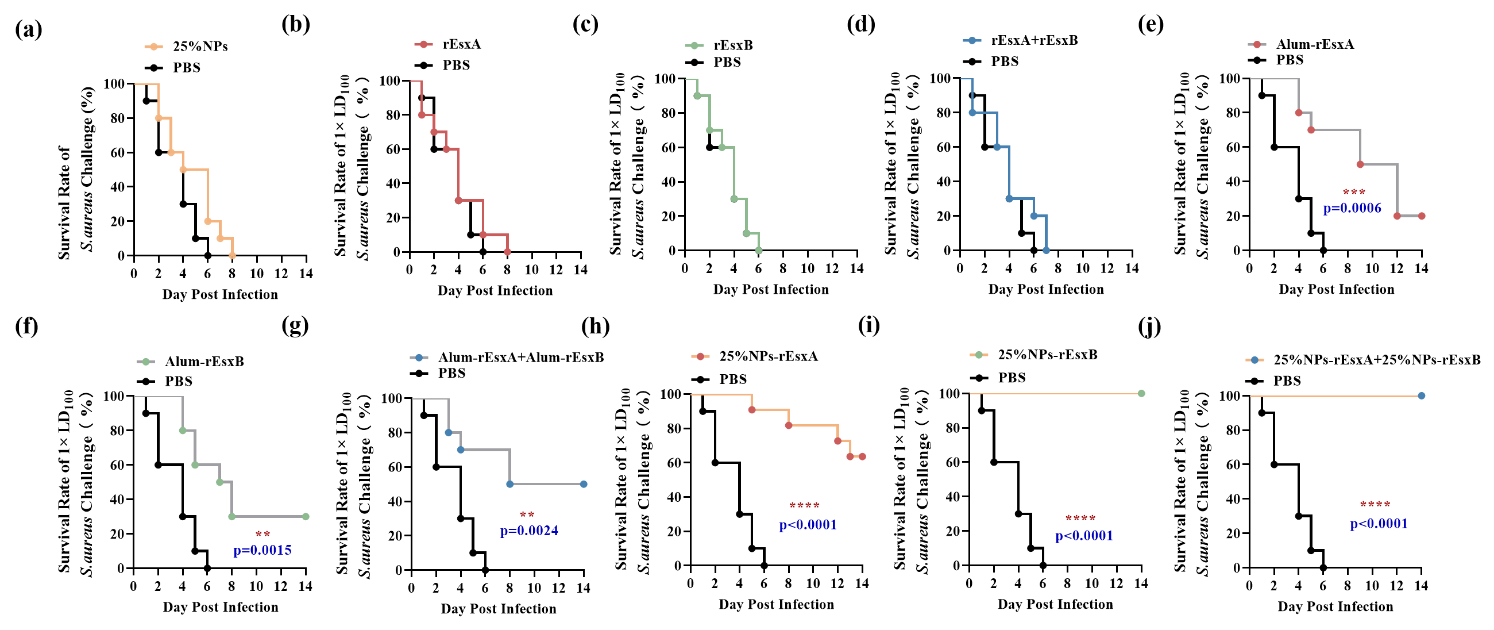


**Supplementary Figure 6.** Protective efficacy of vaccinated BALB/c mice after *S. aureus* challenge. (**a**-**k**) Survival rate (n = 10) of the immunized mice were challenged with *S. aureus* ATCC 25923 strain (5.26 × 10^8^ CFU) by I.V. injection. Survival rates were analyzed with Log-rank (Mantel-Cox) analysis. A *p* value < 0.05 was considered as statistically significant (* *p* < 0.05; ** *p* < 0.01; *** *p* < 0.001; **** *p* < 0.0001).


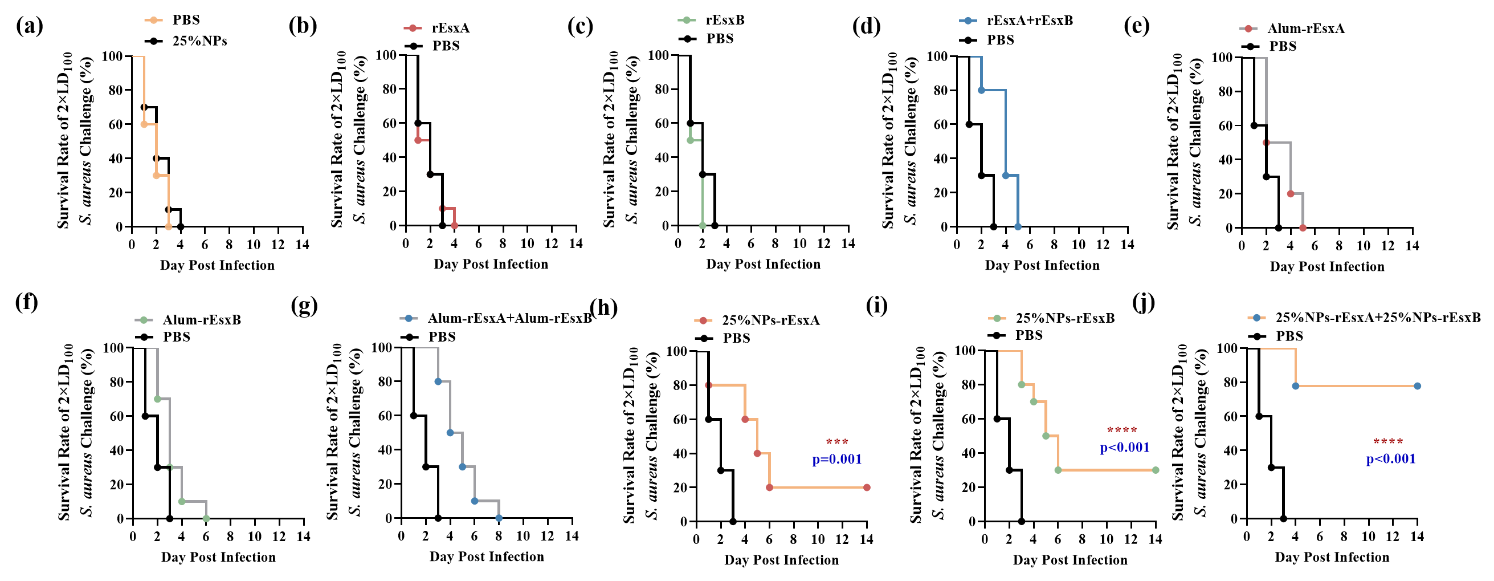


**Supplementary Figure 7.** Protective efficacy of vaccinated BALB/c mice after *S. aureus* challenge. (**a**-**j**) Survival rate (n = 10) of the immunized mice were challenged with *S. aureus* ATCC 25923 strain (10.52 × 10^8^ CFU) by I.V. injection. Survival rates were analyzed with Log-rank (Mantel–Cox) analysis. A *p* value < 0.05 was considered as statistically significant (* *p* < 0.05; ** *p* < 0.01; *** *p* < 0.001; **** *p* < 0.0001).
